# Advancing 3D Dental Implant Finite Element Analysis: Incorporating Biomimetic Trabecular Bone with Varied Pore Sizes in Voronoi Lattices

**DOI:** 10.3390/jfb15040094

**Published:** 2024-04-04

**Authors:** Dawit Bogale Alemayehu, Masahiro Todoh, Song-Jeng Huang

**Affiliations:** 1 Division of Human Mechanical Systems and Design, Graduate School of Engineering, Hokkaido University, Sapporo 060-8628, Japan; zetseatdawit2018@gmail.com; 2 Division of Mechanical and Aerospace Engineering, Faculty of Engineering, Hokkaido University, Sapporo 060-8628, Japan; todoh@eng.hokudai.ac.jp; 3 Department of Mechanical Engineering, National Taiwan University of Science and Technology, Taipei 10607, Taiwan

**Keywords:** finite element analysis, Voronoi lattice, dental implant, trabecular bone, human mandible’s, nTopology, creo parametric, computer aided design, porosity

## Abstract

The human mandible’s cancellous bone, which is characterized by its unique porosity and directional sensitivity to external forces, is crucial for sustaining biting stress. Traditional computer- aided design (CAD) models fail to fully represent the bone’s anisotropic structure and thus depend on simple isotropic assumptions. For our research, we use the latest versions of nTOP 4.17.3 and Creo Parametric 8.0 software to make biomimetic Voronoi lattice models that accurately reflect the complex geometry and mechanical properties of trabecular bone. The porosity of human cancellous bone is accurately modeled in this work using biomimetic Voronoi lattice models. The porosities range from 70% to 95%, which can be achieved by changing the pore sizes to 1.0 mm, 1.5 mm, 2.0 mm, and 2.5 mm. Finite element analysis (FEA) was used to examine the displacements, stresses, and strains acting on dental implants with a buttress thread, abutment, retaining screw, and biting load surface. The results show that the Voronoi model accurately depicts the complex anatomy of the trabecular bone in the human jaw, compared to standard solid block models. The ideal pore size for biomimetic Voronoi lattice trabecular bone models is 2 mm, taking in to account both the von Mises stress distribution over the dental implant, screw retention, cortical bone, cancellous bone, and micromotions. This pore size displayed balanced performance by successfully matching natural bone’s mechanical characteristics. Advanced FEA improves the biomechanical understanding of how bones and implants interact by creating more accurate models of biological problems and dynamic loading situations. This makes biomechanical engineering better.

## 1. Introduction

The complex architecture of human bone, particularly the cancellous or spongy bone in the jaw, is a significant challenge to dental implant design and biomimetic engineering. As a result, the field of study has implemented advanced modeling techniques, with Voronoi lattice structures being widely preferred [1,2,3,4]. Notorious for their distinct open-cell structure that sets them apart from other lattice families, like triply periodic minimal surface (TPMS) gyroid and sheet-type lattices, Voronoi lattices are recognized for their remarkable ability to precisely resemble the intricate and porous properties of human bone [5,6,7,8,9,10,11]. Voronoi lattices are made up of a network of irregular, mathematically determined cell shapes that are very different in how they change shape and how well they can handle mechanical forces. Thus, making them extremely advantageous for scaffolding applications in the field of biomedical engineering. These scaffolds improve osseointegration and tissue ingrowth in addition to mimicking the natural porosity of bone, which usually varies from 60% to 90% [12,13,14,15,16,17]. Because of this, Voronoi lattices are necessary for making biomimetic implants because they offer a customized method that is better at copying the complex anatomical and functional details of human bone than other types of lattices [18,19,20,21,22].

Similar to composite materials, bone has directional dependency under external loads [23,24,25,26,27,28], which makes it crucial yet challenging to accurately simulate using traditional computer-aided design (CAD) models [29,30,31]. These conventional models, which frequently assume isotropic or orthotropic features, considerably simplify the complex behavior of human cancellous bone. When compared to actual clinical dental scenarios, this simplification significantly reduces the accuracy of computational simulations [32]. The majority of earlier research has resorted to replacing human cancellous bone in finite element analysis with solid cross-section block CAD models, a technique that severely reduces the precision of numerical approximations [31,32,33,34,35,36,37]. Voronoi lattice structures provide a remedy to this problem due to their capacity for customized design. They make it possible to adjust Young’s modulus such that it more nearly resembles the modulus of common implant materials, including titanium and its alloys. This tailoring facilitates the mechanical synergy that is necessary for dental implants to integrate effortlessly and remain stable over time inside the human mandible. By strategically using Voronoi lattices, researchers can potentially guarantee a better level of accuracy and clinical relevance in implant design by bridging the gap between simplified assumptions and the complicated nature of biomechanical behavior.

The main focus of this work is to study biomimetic Voronoi lattice structures, which are unique in that they can mimic the porous properties of trabecular bone, which is an important part of human cancellous bone. The porosity of human cancellous bone, which usually ranges from 30% to 90%, is noteworthy and significant for its biomechanical function [17,38,39,40]. The Gibson–Ashby theory demonstrates the significance of the porosity and relative density in defining the mechanical strength and stiffness of cancellous bone by relating the mechanical properties and physical structure of porous materials [5,41,42,43,44,45]. This theory demonstrates how the relative density of cancellous bone, which is a measure of its structural solidity against porosity, directly affects its biomechanical properties. This is how Voronoi lattice structures are different from other types of lattices, like sheet-type lattices, because they can perfectly replicate the unevenly distributed, networked porosity of cancellous bone. This feature improves the biomechanical reliability of finite element analysis (FEA) simulations in addition to promoting osseointegration. 

The finite element method (FEM) is used in this study to improve the mechanobiology analysis of dental implants [1,20,21,24,29,30,31,32,34,35,36,37,44,46,47,48,49]. FEM is a well-known computer method for modeling and analyzing complex physical events by breaking a continuum into a few discrete parts. The use of finite element method (FEM) in biomechanics and biomimetics has transformed the study of cancellous bone structures by providing a comprehensive understanding of how they behave under different stress scenarios [50,51,52,53,54,55,56]. In particular, this work uses FEM to simulate dynamic, slow loading conditions that are precisely designed to mimic the cyclic biting forces applied to dental implants during mastication. This robust approach differs from earlier research that mostly relied on static loading assumptions, ignoring the dynamic and cyclical nature of mandibular stress during chewing [30,53,57,58,59,60,61,62]. By using smooth step-time convergence, this study is more like the explicit dynamic loading that happens in clinical settings. It gives a more accurate and complete look at the stress, strain, and displacement that happen in dental implants. This improved modeling method provides insight into the mechanical behavior of the implants over time, which is a major advancement over previous static loading assumptions. Previous studies have highlighted the shortcomings of static models in representing the complex biomechanical environment of the jaw [32,35]. These studies have advocated for the incorporation of dynamic loading parameters in order to more accurately simulate the physiological conditions of mastication.

This study carefully assesses how differences in pore sizes in biomimetic Voronoi lattice structures affect their capacity to bear external stresses, with particular emphasis on improving dental implant biomechanics. An adequate pore size not only increases the load-bearing capacity of the implant but also promotes an ideal biomechanical environment by reducing stress-shielding effects and enabling uniform stress distribution [63]. In order to acquire mechanical properties that match those of natural bone, pore size modifications are strategically used to control porosity and relative density [64,65,66]. This promotes osseointegration and improves the long-term durability of the implant. The mechanical integrity and biological performance of implants are greatly influenced by pore sizes within a certain range [67,68,69]. These studies show that increased porosity often results in decreased mechanical strength, even if it is advantageous for biological integration. Nonetheless, a tailored approach that maximizes the strength-to-weight ratio while maintaining biological functioning is made possible by the use of Voronoi lattice structures, which are in line with cancellous bone’s inherent properties [70].

By using FEA, the main goal of this study is to improve the comprehension of the biomechanical properties of dental implants. In particular, it focuses on using biomimetic Voronoi lattice architectures with different pore diameters to effectively mimic human cancellous bone characteristics. In order to precisely replicate masticatory conditions seen in real life, every component of the dental implant is thoroughly examined in the present study. It also explores into effects to the implant parts and the trabecular bone mimic when the pore size changes in the Voronoi lattice. Thus, this work anticipates that the novel Voronoi lattice cancellous bone pore size changes have a significant impact on the lattice’s relative density and porosity, which are required to replicate the integrity and strength of real cancellous bone. This work clarifies the crucial role that structural changes play in varying the overall biomechanical effectiveness of dental implant systems through a thorough investigation of biomechanical parameters—displacements, stresses, and strains—derived from the FEA.

## 2. Materials and Methods

### 2.1. 2D CAD Drawing and Dimensions

**Figure 1:**
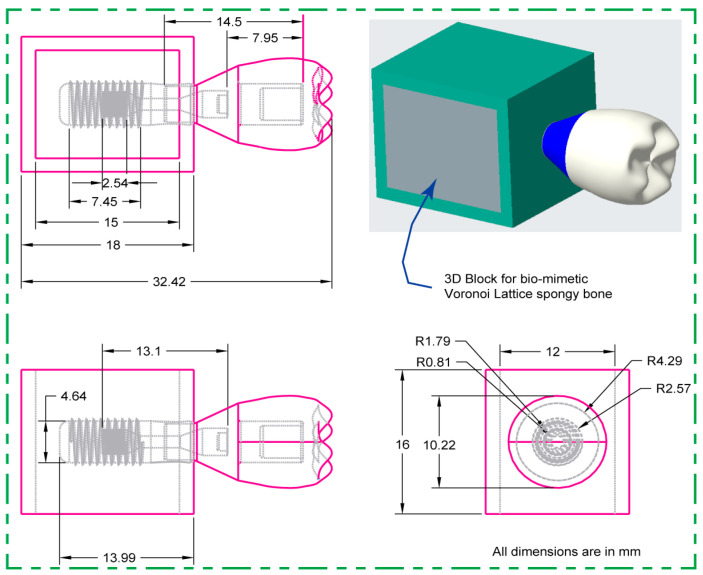
Two-dimensional drawing of dental implant assembly in creo parametric: front, side, and top views with detailed dimensions (mm).

The dental implant system’s main parts—cancellous and cortical bone structures—were rigorously designed with the use of Creo Parametric 8.0 software. As seen in Figure 1, this method constructed both two-dimensional sketches and three-dimensional designed models with precise dimensions.

### 2.2. Pore Sizes and Voronoi Latticed Bone

**Figure 2:**
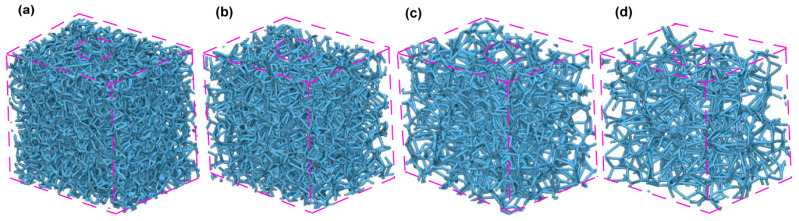
Comparative analysis of biomimetic Voronoi latticed spongy bone structures with four distinct pore sizes, (**a**) 1.0 mm, (**b**) 1.5 mm, (**c**) 2.0 mm, and (**d**) 2.5 mm, designed using nTopology (nTop 4.17.3) software.

Compact bone is defined by a relatively low porosity, usually less than 15%, while certain instances show a porosity range of around 5–30% [71]. On the other hand, trabecular bone, or cancellous bone, has much greater porosity; it often exceeds 70%. However, there are variances ranging from around 30% to over 90% [72]. This trabecular structure has a relative density that varies between 0.05 and 0.3, indicating that it is very cellular [73]. Based on previously published data on the natural porosity of human trabecular bone, this work uses nTopology software (nTOP 4.17.3) to generate four biomimetic Voronoi lattice models with pore sizes of 1.0 mm, 1.5 mm, 2.0 mm, and 2.5 mm. These models, known as Voronoi Trabecular Bone (VTB) structures, are designated VTB10, VTB15, VTB20, and VTB25, respectively. Figure 2 shows these four models with different pore sizes. Using finite element analysis (FEA), the study precisely investigates the impact of these various pore sizes in the Voronoi lattice structure on the stresses and strains that bones and other dental implant components experience. The objective of this methodology is to better understand the biomechanical effects of pore size variations on implant stability and bone stability.

### 2.3. Building, FE Volume Meshing, and Boundary Condition of Voronoi Lattice

**Figure 3:**
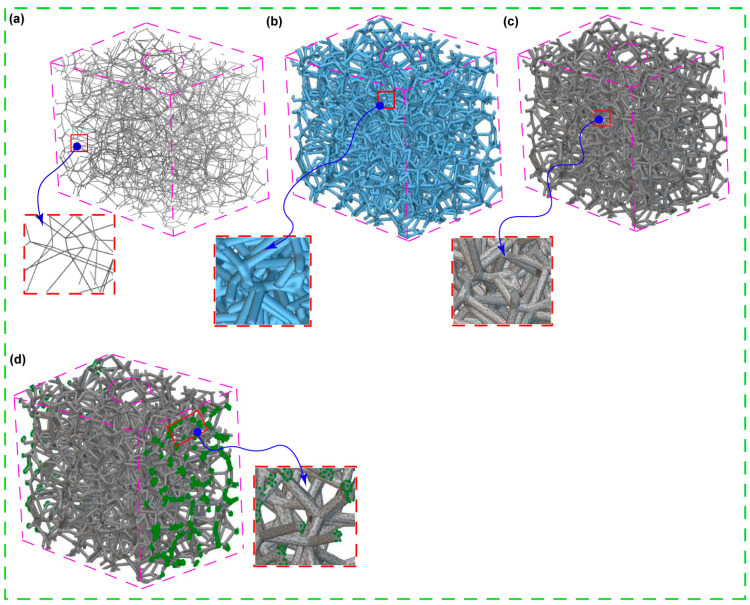
Biomimetic Voronoi-latticed trabecular bone constructed in nTopology featuring the following: (**a**) open-cell randomized lattice graph, (**b**) truncated thicken lattice graph, (**c**) finite element (FE) volume mesh, (**d**) FE boundary configuration utilizing body nodes. Each red squares with blue arros are the zoomed and detailed view for clarity purpose of images from (**a**–**d**).

A solid 3D CAD model saved as a Standard ACIS Text (SAT) file is imported and used as the design area to create Voronoi-latticed models that resemble the cancellous bone structure. Then, using a randomization seed of 250 and four different pore sizes as shown in Figure 2, a randomized lattice graph is produced (as shown in Figure 3a). The lattice graph is thickened with a beam diameter of 0.3 mm to strengthen the structure. In order to reduce structural complexity and remove unnecessary overhangs, a lattice trimming procedure is used with a feature tolerance of 10. By exercising precision, a biomimetic scaffold that closely mimics the architecture of cancellous bone could be designed, allowing for a possible exploration of its biomechanical properties.

### 2.4. Oblique Load and FE Boundary Conditions

**Figure 4:**
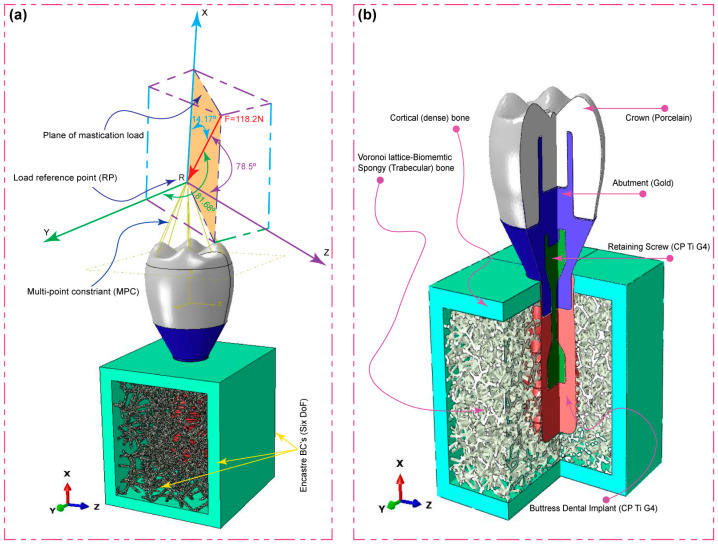
Components of the dental implant system, including the following: (**a**) application of dynamic explicit oblique loading at the load reference point with the MPC constraint, (**b**) 3D model components designed for finite element analysis.

As shown in Figure 4a, the occlusal crown surface was loaded with forces in three different directions: mesiodistal, buccal-lingual, and apical. This is to illustrate the biomechanical response of the dental implant under multi-axial dynamic oblique loading. There were three different specific force magnitudes applied: 23.4 N, 17.1 N, and 114.6 N. By using a multi-point constraint (MPC) approach, these forces converged at a dummy reference point that was 3 mm from the occlusal surface. This method replicated the complex forces encountered during clinical mastication by generating an equivalent force of 118.2 N, tilted at 75.8 degrees with respect to the occlusal plane [32,34,35,36,74].

To be more specific, the simulation method included explicit dynamic loading at a slower rate to mimic the cyclic mastication stress seen in real life. Notably, to accurately mimic in vivo situations, the load was applied over a 0.5 s period in the dynamic scenario, representing the typical mastication frequency of 2 Hz [32,34,75]. Specially designed boundary conditions were used to mimic real-life constraints. At the implant–mandible interface, a six-degree-of-freedom (DoF) Encastre boundary condition was used to fix the implant in all three spatial dimensions (X, Y, and Z). The objective of this configuration was to accurately replicate the distribution of combined stress in the mesiodistal, buccal-lingual, and apical directions. Finite Element (FE) Boundary by Body Modeling was used to represent the biomimetic Voronoi cancellous bone structure. As shown in Figure 2, this technique made it easier to accurately export nodes from nTop for boundary condition assignment in the ABAQUS/CAE 2023 software environment. All dental implant constituents were investigated, including the artificial Voronoi lattice cancellous bone (Figure 4b). The study accurately measured the compressive and tensile stresses in the implant system using finite element analysis with ABAQUS. This makes progress in the study of implant biomechanics under physiologically important loading scenarios.

### 2.5. Physical Properties and FE Mesh

**Figure 5:**
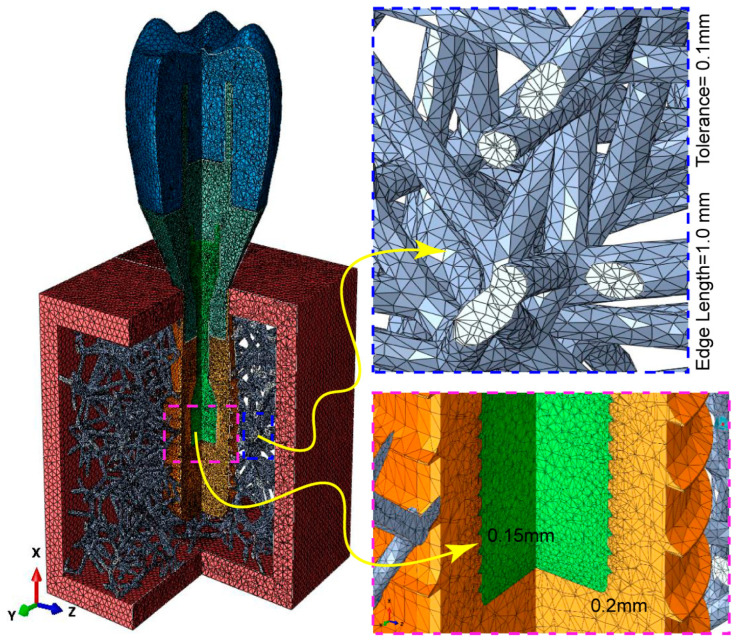
Detailed meshing of dental implant components and Voronoi lattice biomimetic bone, with an emphasis on the mesh configuration, as illustrated in the zoomed section.

It is very important to mesh the model properly so that there are fewer numerical errors and the results of finite element analysis (FEA) are more reliable when compared to real-world events. Using nTopology (nTOP 4.17.3) software, the Voronoi cancellous bone meshing was performed consecutively in the present study. First, an implicit body was constructed from a traditional 3D solid CAD model of spongy bone that was imported from Creo Parametric software in order to utilize it as a design space. This implicit design area was then used for building a Voronoi lattice, which was then utilized for lattice meshing. Robust tetrahedral meshing was used after surface re-meshing steps to address the structure’s complexity. Figure 5 serves as an illustration of the choice of an edge length of 1.0 mm and a feature tolerance of 0.25 mm for this purpose. To prepare the model to use finite element analysis, the last step was performing FE volume meshing. To be more precise, several element sizes were used to mesh the dental implant components: 0.2 mm for the implant, 0.15 mm for the screw-retaining components, and 0.35 mm for the remaining components [32]. The meshing procedure was carried out using ABAQUS software (see Figure 5). The FE mesh statistics for all the implant system components are presented in Table 1. The physical characteristics of the implant and bone materials are presented in Table 2. It is assumed that the components of the implant, comprising the screw, abutment, and crown, have homogeneous, elastic characteristics.

### 2.6. Relative Density and Porosity of Biomimetic Voronoi Lattice

The nTopology (nTop 4.17.3) program is used to make a Voronoi-latticed biomimetic bone structure, which is different from traditional modeling methods. With this method, the surface area-to-volume ratio, relative density, and porosity of the lattice structure can all be accurately calculated. Understanding the mechanical characteristics and potential for the osteointegration of biomimetic bones requires knowledge of these analyses. The following equations provide a mathematical basis for these key parameters:

Relative Density ($\rho_{rel}$) of Biomimetic Bone:

(1) $$\rho_{rel}=\frac{M_{La}}{M_{Sol}}$$

Surface Area-to-Volume Ratio (*SA/V*) of Voronoi Lattice:

(2) $$\;SA/V=\frac{A_{La}}{V_{La}}$$

Porosity (*Φ*) Relating to Relative Density:

(3) $$\Phi =1-\rho_{rel}$$

where $M_{La}$ is the mass of the lattice structure, $M_{Sol}$ is the mass of the solid (CAD model) volume, $A_{La}$ is the total surface area of the lattice, and $V_{La}$ is the total volume of the lattice.

## 3. Results

### 3.1. Voronoi Lattice Pore Size

Models with four different pore sizes—1 mm, 1.5 mm, 2 mm, and 2.5 mm—were created using nTopology (nTOP 4.17.3) software, all of which had a constant 0.4 mm beam thickness. The influence of these variations in pore size on a variety of essential lattice structure parameters, such as the average beam length, number of beams, number of nodes, relative density, and surface-area-to-volume ratio, was well established by our analysis (see Table 3). The study found a significant pattern associated with an increasing pore size. Notably, the average beam length rose as the pore size increased, from 0.4614 mm at 1 mm to 0.9203 mm at 2.5 mm, showing the need for longer beams to cross the increasing distances between nodes. From the smallest to the biggest pore diameters, the lattice beam and node counts significantly decreased simultaneously with increasing pores, falling from 25,809 to 3024 and 15,186 to 2058, respectively. This reduction allows for bigger spaces of voids by signaling a decline in the lattice complexity and density. Additionally, a clear decrease in relative density was seen, decreasing from 22.21% for the 1 mm pore size to 6.27% for the 2.5 mm pore size. This indicates that pore size and lattice density are inversely related.

### 3.2. Pore Size versus Relative Density and Porosity

**Figure 6:**
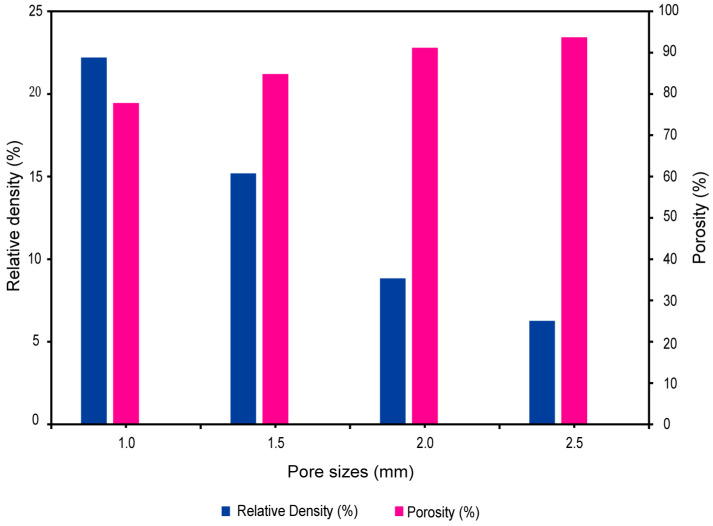
Relative density and porosity of biomimetic Voronoi lattice trabecular bone across various pore sizes.

The range of pore sizes in biomimetic Voronoi lattice structures, shown in Figure 6, from 1.0 mm (VTB10) to 2.5 mm (VTB25), is an important factor for describing the pore scale and has a direct impact on the relative density and porosity of the scaffold. The relative density, which measures the lattice structure’s compactness in comparison to its solid equivalent, steadily drops with an increasing pore size; it declines from 22.21% at 1.0 mm to 6.27% at 2.5 mm. This trend represents a gradual decline toward a structure that is less dense. On the other hand, porosity measures the volume percentage of empty space within the lattice and has an inverse relationship with relative density. With increasing pore sizes, Figure 6 shows an increasing porosity trend that ranges from 77.79% for VTB10 to 93.73% for VTB25.

### 3.3. Dynamic Oblique Loading

**Figure 7:**
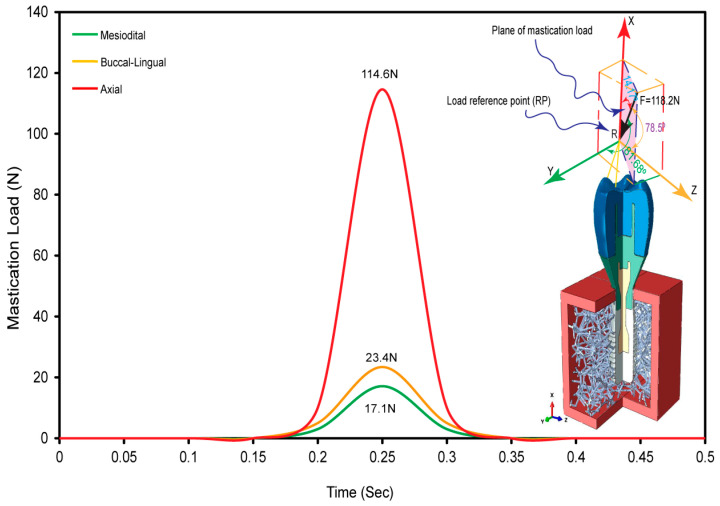
An equivalent oblique dynamic load distribution on the dental implant crown during mastication, a multidirectional analysis over 0.5 s cycle.

Figure 7 displays the application of an oblique dynamic load of 118.2 N to the occlusal surface of the crown. The load is directed in the buccal–lingual, axial, and mesiodistal directions, simulating a 2 Hz mastication cycle with a plane of mastication load. The figure shows the smooth change in load on the crown’s occlusal surface across mesiodistal, buccal–lingual, and apical orientations throughout a 0.5 s mastication cycle. The goals of this study were met by using multi-point constraints (MPCs) and a reference point as the master control to create a smooth step amplitude on the occlusal surface of the crown. The load distribution was in the three designated directions, which matches the findings of a previous study [32]. This proves that the masticatory forces on dental implants that were simulated in this research method are accurate.

### 3.4. Von Mises Stress in Dental Implant Assembly

**Figure 8:**
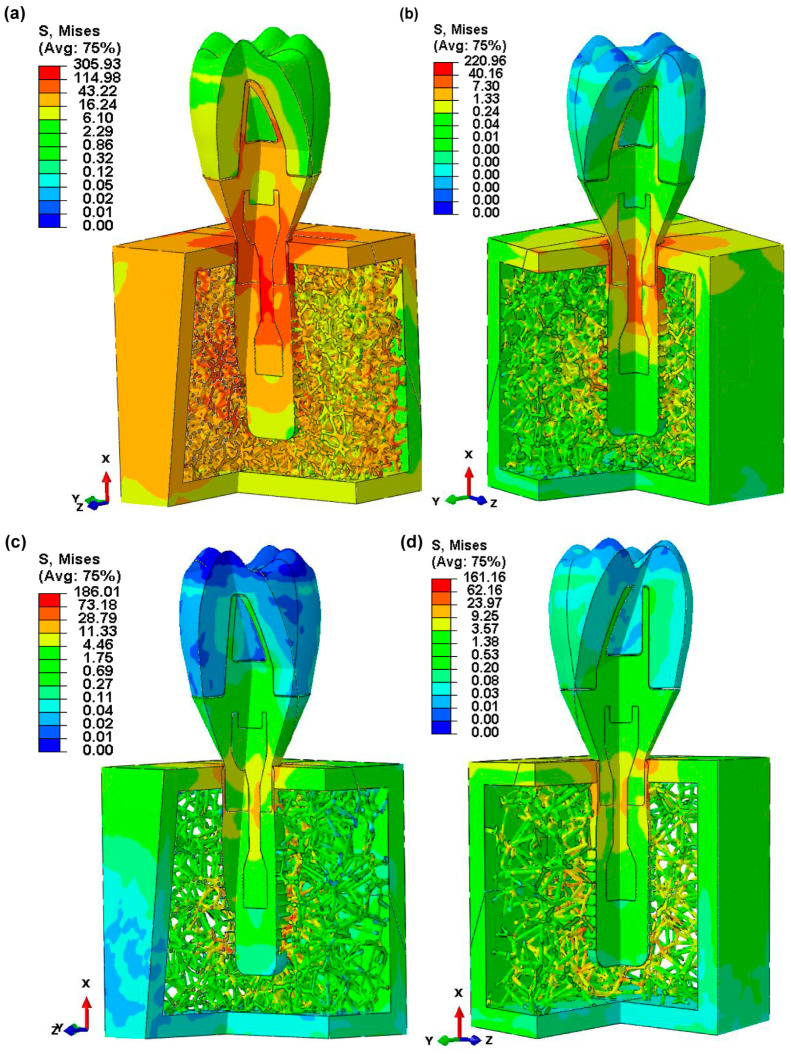
Maximum von Mises stress distribution in double-sliced assembled implant parts for four biomimetic Voronoi-latticed trabecular bones: (**a**) VTB10, (**b**) VTB15, (**c**) VTB20, and (**d**) VTB25.

Figure 8 depicts the maximum von Mises stress values for a doubly sliced assembly of dental implant components, exhibiting stress distributions across four different biomimetic Voronoi lattice trabecular bone designs. The findings show a decreasing trend in the von Mises stress with an increasing pore size. The stress values are reported as 305.93 MPa for a 1 mm pore size (VTB10), subsequently decreasing to 220.96 MPa for 1.5 mm (VTB15), 186.01 MPa for 2 mm (VTB20), and ultimately to 161.16 MPa for 2.5 mm (VTB25).

### 3.5. Von Mises Stress in Dental Implant and Retaining Screw

**Figure 9:**
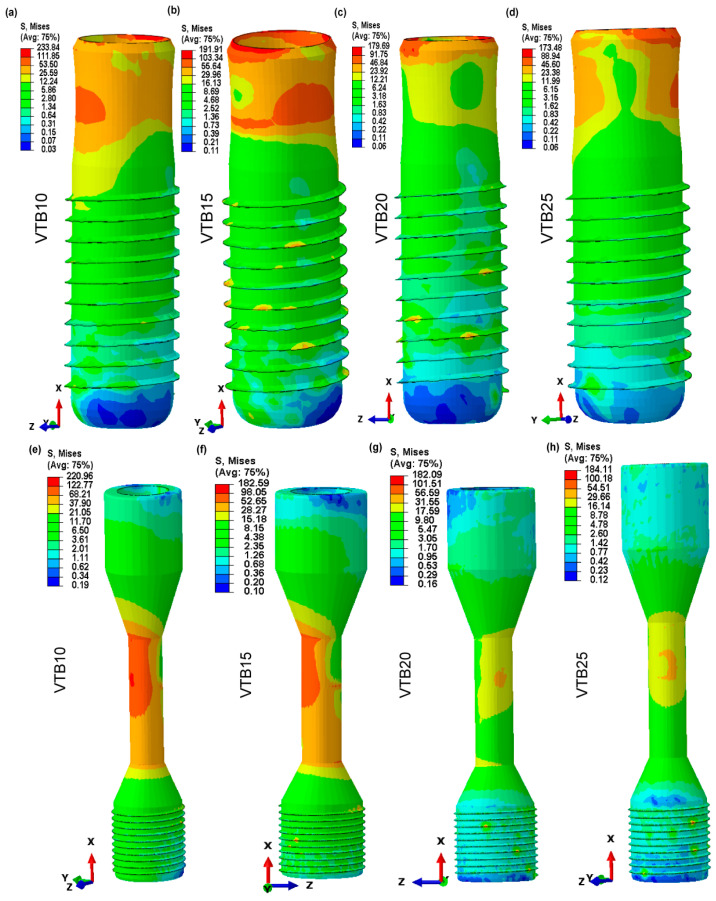
Contour von Mises stress distribution in dental implants and retaining screws across biomimetic Voronoi-latticed trabecular bone pore sizes: (**a**) VTB10, (**b**) VTB15, (**c**) VTB20, (**d**) VTB25 for implants, and (**e**) VTB10, (**f**) VTB15, (**g**) VTB20, (**h**) VTB25 for retaining screws.

The von Mises stress analysis, shown in Figure 9a–h, illustrates the effect of various pore sizes inside the biomimetic Voronoi-latticed trabecular bones on stress distribution in both the dental implant and the retaining screw. The von Mises stress for the dental implant consistently decreases as the pore size increases, from 1 mm (VTB10) to 2.5 mm (VTB25), falling from 233.84 MPa to 173.48 MPa, respectively. The retaining screw has an identical trend but a less noticeable decline from 220.96 MPa to 184.11 MPa. Interestingly, the 2 mm pore size (VTB20) indicates a key threshold where the implant’s von Mises stress significantly decreases to 179.69 MPa, which is in close alignment with the retaining screw’s stress levels (182.09 MPa).

### 3.6. Von Mises Stress in Cortical and Voronoi Trabecular Bone

**Figure 10:**
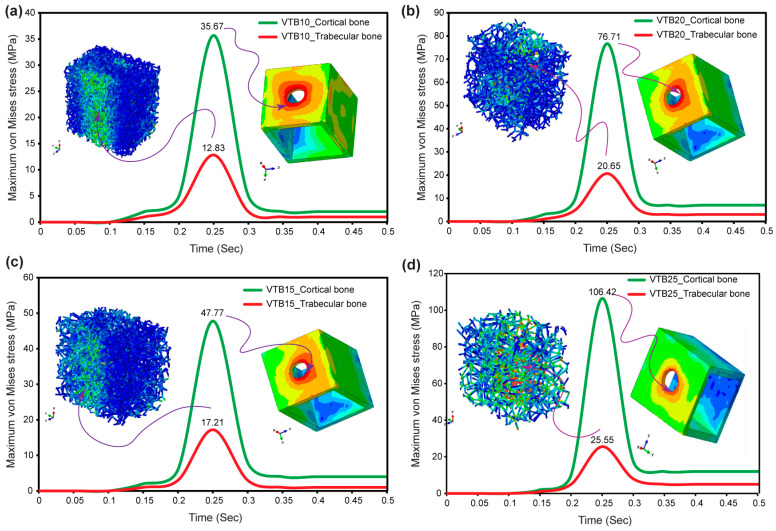
Maximum von Mises stresses in biomimetic Voronoi lattice trabecular and cortical bones for (**a**) VTB10, (**b**) VTB15, (**c**) VTB20, and (**d**) VTB25.

The results from Figure 10a–d, combined with observed stress concentration patterns, provide a comprehensive understanding of maximum von Mises stress behavior in both cortical and biomimetic Voronoi lattice trabecular bones under a mastication frequency of 2 Hz, corresponding to a cycle time of 0.5 s. Notably, regions of possible mechanical risk are indicated by the concentration of the highest stress around the hole where the cortical bone contacts the implant. Stress distribution inside the Voronoi lattice trabecular bone is consistent throughout different pore sizes, ranging from 12.83 MPa at 1.0 mm (VTB10) to 25.55 MPa at 2.5 mm (VTB25). Likewise, stress in the cortical bone increases from 35.67 MPa (VTB10) to 106.42 MPa (VTB25) as the trabecular structure pore size increases, indicating a clear relationship between the pore size and stress effect on both types of bone. Stress levels peak at about 0.35 s into the mastication cycle, remaining constant even as cycles continue.

### 3.7. Micromotions in Voronoi Trabecular Bone

**Figure 11:**
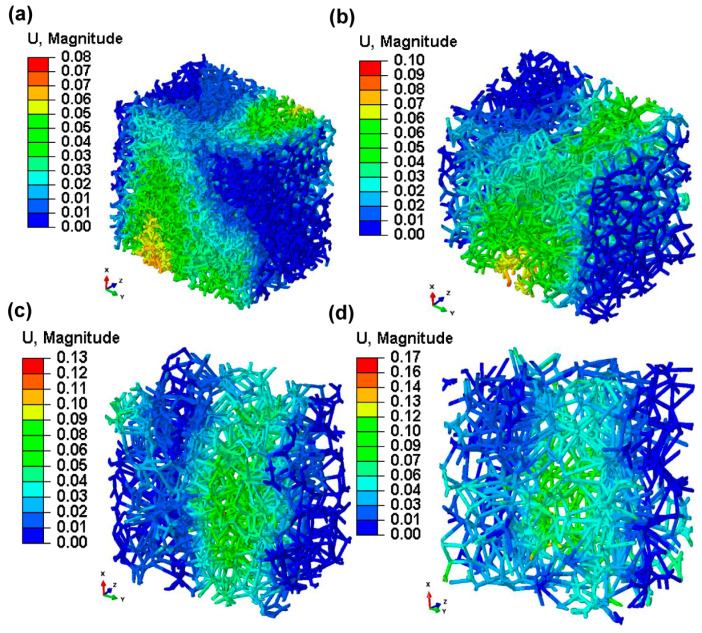
Contour plots of the magnitude of displacements in biomimetic Voronoi lattice trabecular bone for pore sizes: (**a**) VTB10, (**b**) VTB15, (**c**) VTB20, (**d**) VTB25.

Figure 11, which depicts contour plots of the magnitude of micromotion inside the biomimetic Voronoi-latticed trabecular bone subjected to finite element analysis, shows a clear relationship between the pore size and micromotion magnitude. Specifically, micromotion values inside the biomimetic Voronoi trabecular bone increased gradually as pore diameters increased. For a pore size of 1.0 mm (VTB10), the micromotion was 8.00 μm. This micromotion increased progressively with the pore size; at 1.5 mm (VTB15), it was reported at 10 μm, rising to 13.00 μm at 2.0 mm (VTB20), and reaching 17.00 μm at the maximum pore size of 2.5 mm (VTB25). These results show how important pore size is when studying the biomechanical behavior of the biomimetic Voronoi trabecular bone, especially micromotion when dynamic mastication stresses are present.

### 3.8. Displacement in Dental Implant Assembly

**Figure 12:**
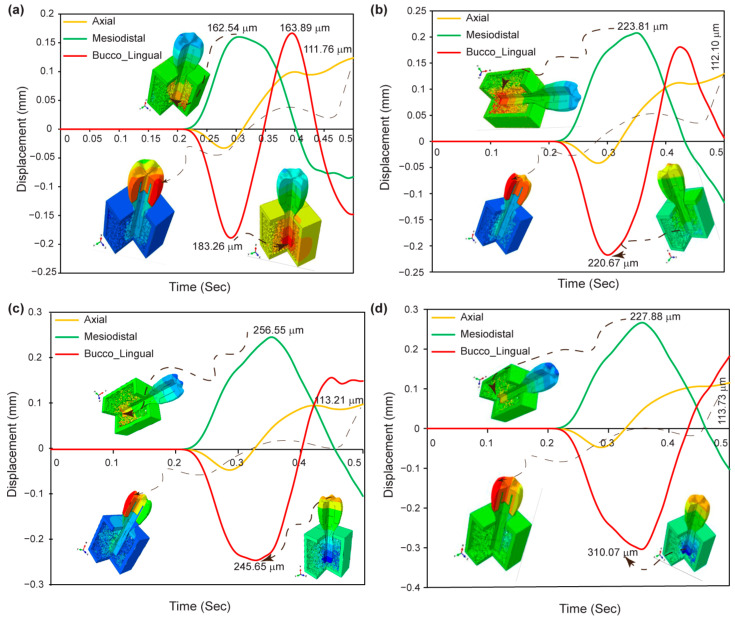
Maximum displacement response in axial, mesiodistal, and buccolingual directions for biomimetic Voronoi lattice trabecular bones: (**a**) VTB10, (**b**) VTB15, (**c**) VTB20, and (**d**) VTB25.

Figure 12a–d shows the maximum displacement found in the axial, mesiodistal, and buccolingual directions for various pore sizes of biomimetic Voronoi-latticed trabecular bone. The findings show that biomimetic Voronoi-latticed trabecular bones with varied pore sizes (VTB10, VTB15, VTB20, and VTB25) exhibit maximal displacements in the axial, mesiodistal, and buccolingual directions. The displacements in the axial direction range from 111.76 μm for VTB10 to 117.73 μm for VTB25. VTB20 has a mesiodistal displacement peak of 256.55 μm, whereas VTB25 has the largest buccolingual displacement at 310.07 μm.

### 3.9. Reaction Forces in Dental Implant Assembly

**Figure 13:**
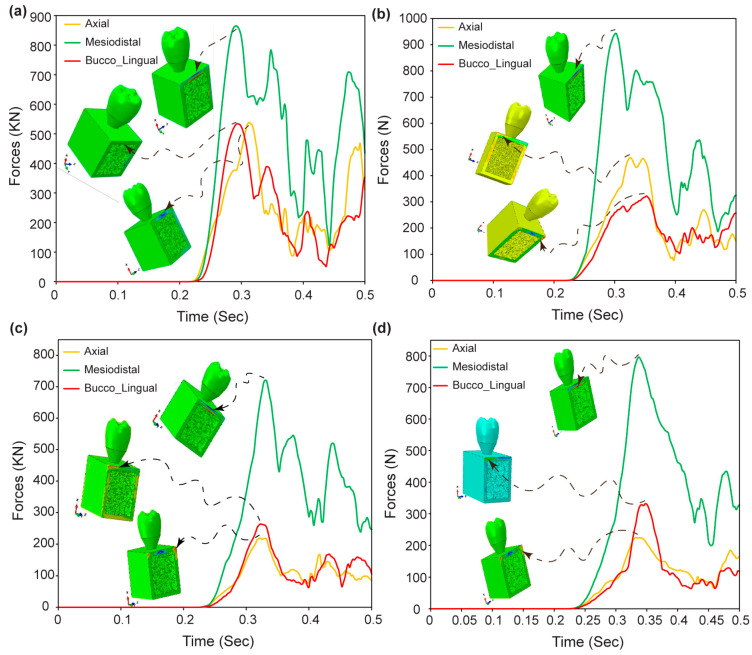
Dynamic reaction forces in dental implant assemblies across biomimetic Voronoi-latticed trabecular bone pore sizes: (**a**) VTB10, (**b**) VTB15, (**c**) VTB20, and (**d**) VTB25.

Figure 13a–d shows the dynamic response forces for the biomimetic Voronoi-latticed trabecular bones with various pore sizes (VTB10, VTB15, VTB20, and VTB25) in the axial, mesiodistal, and buccolingual directions. Initially, response forces are zero across all models until 0.23 s, after which they increase in line with the dynamic loading amplitude. After 0.23 s, the maximum response forces are observed: VTB10 has forces of 529.41 KN axially, 858.6 KN mesiodistally, and 530.86 KN buccolingually; VTB15 has 465.09 KN, 927.27 KN, and 316.98 KN, respectively; VTB20 has 215.25 KN, 717.68 KN, and 259.8 KN; and VTB25 has 224.21 KN, 788.69 KN, and 329.24 KN in the respective directions.

## 4. Discussion

This study looked into the biomechanical design of dental implant systems in great detail. It used cutting-edge software, like Creo Parametric and nTopology (nTop 4.17.3), to accurately model both cancellous and cortical bone structures. We used advanced modeling techniques to make biomimetic Voronoi lattice structures with different pore sizes that are very similar to the natural porosity and mechanical properties of human bone. We used finite element analysis (FEA) to assess the impact of these pore sizes on stress and strain distributions within the bone and implant components, aiming to enhance implant stability and osseointegration. The main results showed that holes with an intermediate size, especially those with a diameter of 2.0 mm, made the implants much more biomechanically compatible. This suggests that dental implant designs and clinical uses could be improved in this way. The findings we obtained support the argument that was proposed in the study’s introduction. It is suggested that the variation in pore size inside biomimetic Voronoi lattice structures affects the stress distribution and micromotion across the dental implant components, as well as the surrounding cancellous bone. According to our investigation, a pore size of 1.5 mm provides optimum equilibrium and closely mimics the dynamic loading response observed in human cancellous bone. This pore size makes the stress distribution significantly uniform at the implant–bone contact (BIC) by balancing structural factors, like the beam length and node count, with the biomechanical needs of dental implants. With a relative density of 15.19% and a surface-area-to-volume ratio that promotes cell attachment and nutrient transport, this option maintains structural strength while also facilitating osteointegration. The design effectively mimics the porosity structure of real bone, improving both mechanical stability and biological compatibility. Because it distributes stress analogously to naturally occurring cancellous bone, it can sustain physiological loads and lower the likelihood of stress shielding and implant failure. Our present findings show that the lattice structure has a porosity of around 84.81%, which closely matches the porosity range of 75% to 90% seen in real human cancellous bone. This discovery is consistent with previous research, which has repeatedly established that trabecular bone has a porosity greater than 70% [17,72,73,76,77,78], highlighting the biomimetic accuracy of our lattice design. This shows that the chosen pore size worked well. This congruence verifies our study’s biomimetic methodology and supports the integration and vascularization that are essential to dental implants’ long-term effectiveness. As such, it highlights the possibility of tailored biomimetic Voronoi lattices to augment the lifetime and efficacy of dental implants, thus making them a model option for mimicking the intricate interplay between strength and porosity that characterizes human cancellous bone. Our findings, which show that a 2.0 mm pore size (VTB20) is better for distributing stress and making dental implants last longer, are in line with what other studies have found. Our findings support previous research showing the efficiency of biomimetic implants in distributing stress to neighboring bone structures, ensuring safety and structural integrity under normal loading scenarios [77]. This agreement underlines the need for regulating pore size to enhance the biomechanical compatibility and performance of dental implants, establishing the foundations for future advances in implant design and application. 

Figure 6 depicts the study’s findings, which show a significant relationship between pore size and the mechanical integrity of biomimetic Voronoi lattice trabecular bone, particularly in the context of dental implants. Pore diameters increased from 1.0 mm to 2.5 mm, resulting in a decrease in the relative density and an increase in porosity. These variations have an immediate influence on the implant’s stress distribution properties under dynamic loading conditions. A lower relative density, which means bigger pores, is more like the natural structure of human cancellous bone. This makes it easier for the dental implant system to spread stress evenly. By mimicking the natural load-bearing function, this advancement promotes osseointegration and lowers the possibility of implant failure. The 2.0 mm (VTB20) configuration stands out as the best candidate among the assessed pore sizes as it maintains a compromise between preserving adequate structural strength with a relative density of 8.84% and having sufficient porosity (91.16%) for biological integration. This pore size successfully mimics the mechanical and structural characteristics of natural cancellous bone, which optimizes stress distribution and ensures the scaffold’s endurance under physiological loading conditions [77], thereby extending the lifespan and functionality of dental implants.

Figure 8 shows that pore size variations in biomimetic Voronoi lattice trabecular bones have a significant impact on stress distribution across dental implant assemblies, particularly around critical interfaces consisting of implant–bone, implant–abutment, abutment–screw retaining, abutment–crown, and implant–retaining screw contacts. Stress concentrations are significantly reduced when the pore size is increased from 1.0 mm (VTB10) to 2.0 mm (VTB20), which allows for a more uniform stress distribution that is more consistent with the behavior of natural bones. Despite its intended purpose of mimicking human cancellous bone, the stiffness inherent in the smaller-pore-size (VTB10) assembly hinders stress homogeneity, boosting the possibility of localized failure. The larger-pore-size (VTB20) assembly makes the implant system more stable and helps it fit together better by improving its mechanical response and closely matching the load-bearing and stress-dissipation properties of natural cancellous bone. This optimization shows how important it is to change the size of the pores in Voronoi lattice structures to find the right balance between stiffness and porosity when trying to make a design that can withstand mechanical loads and mimic the biological activity of bone tissue.

Figure 9a–h shows that the maximum von Mises stress inside dental implants and their retaining screws is significantly influenced by pore size in biomimetic Voronoi-latticed trabecular bone designs. As the pore size increases from 1 mm (VTB10) to 2.5 mm (VTB25), the research shows a steady drop in von Mises stress values in both the implant and the retaining screw. Notably, the stress in the retaining screw decreased from 220.96 MPa to 184.11 MPa, and the stress in the dental implant decreased from 233.84 MPa for VTB10 to 173.48 MPa for VTB25. According to this trend, larger pores may help distribute stress uniformly, which could prolong the implant system’s lifespan and improve its mechanical stability. Notably, the ideal stress distribution, defined by a compromise between mechanical integrity and biomimetic porosity, was seen for VTB20, demonstrating that a pore size of 2.0 mm is the best for imitating the mechanical characteristics of human cancellous bone. The stress concentration at the implant’s neck and the screw’s mid-region—areas that are often prone to failure or fracture under cyclic dynamic loading—is significantly reduced by this improved design. This adaptation mimics human bone’s inherent load-bearing characteristics, with the potential to increase the implant lifetime and function in clinical conditions. This work strongly corresponds with previous research aims, highlighting the importance of mechanical strength and biological characteristics in titanium materials for biomedical applications [79,80].

Figure 10a–d shows the maximum von Mises stress across cortical and biomimetic Voronoi lattice trabecular bones, emphasizing the critical role of pore size in the biomechanical response of different bone types under masticatory stress. As illustrated, the pore size directly affects the structural integrity and mechanical behavior of the bone–implant system. The even stress distribution inside the Voronoi trabecular structure and the stress concentration at the cortical bone–implant interface serves as evidence for this. The fact that von Mises stress goes up as pore sizes get bigger and stays the same after 0.35 s shows that the structural design and dynamic loading response are complicatedly linked. In particular, changing the size of the pores in the Voronoi lattice trabecular bone creates a biomechanical environment that is similar to how bones naturally behave. This makes the stress distribution better and reduces stress concentrations in certain areas that could cause failure. This optimized distribution pattern not only demonstrates the biomimetic structure’s capacity to imitate the physiological stress of human bones, but it also represents a major improvement in finite element analysis (FEA) results. The biomimetic Voronoi lattice is a better way to predict how bone–implant assemblies will act in real life. This is because it changes the size of the pores to find a balance between biological activity and mechanical support. This study uses biomimetic Voronoi-latticed cancellous bone for finite element analysis, which is very different from earlier studies that used solid CAD blocks to model bone structures. By adding the Voronoi lattice’s detailed, porous design, this study provides a more physiologically realistic description of bone mechanics, improving the predictability of stress distribution patterns under masticatory stresses. This method not only improves the biomechanical modeling of bone–implant interfaces, but it also gives a more nuanced knowledge of the effect of pore size on structural integrity and mechanical behavior. In line with what other studies have found [81,82,83,84], this discovery opens the door to the creation of bone scaffolds and implants that improve healing by closely imitating the properties of natural bone.

Figure 11 shows the sizes of micromotions in biomimetic Voronoi-latticed trabecular bones with four different pore sizes. This shows how important micromotion is in the biomechanics of dental implants. Larger pore diameters are linked with increased micromotion, which increases the risk of bone resorption at the bone–implant interface and fractures in dental implant components, particularly the retaining screw and the implant itself. Additionally, micromotions increase the likelihood of corrosion, especially when the implant works in a biofluid environment. Despite these difficulties, the research finds that a pore size of 2.0 mm (VTB20) with a micromotion of 13 μm provides an ideal compromise between reducing unfavorable biomechanical consequences and providing a satisfactory degree of mechanical rigidity. This pore size facilitates the structural and functional integration of the implant into the biological system while also reducing the chance of implant system failure. As such, these micromotion optimization insights provide an in-depth understanding of the relationship between pore size and implant function, which considerably advances the field of biomechanical dentistry research.

Incorporating the observed displacement patterns from Figure 12a–d, the axial and buccolingual directions show both negative and positive peak displacement amplitudes, indicating that the dental implant can withstand cyclic masticatory stress. The scenario demonstrates the implant’s flexibility and mechanical resilience, which are essential for enduring numerous stress cycles while maintaining structural integrity. Such displacement patterns indicate that VTB20, with its nuanced response in all measured directions, not only mimics the normal biomechanical behavior of human bone, but also guarantees the implant’s capacity to tolerate dynamic stresses experienced in vivo. This modification emphasizes just how essential it is to choose the right pore size in the Voronoi lattice trabecular bone in order to maximize the performance of dental implants. VTB20 offers an excellent balance between mechanical rigidity and flexibility, which is crucial for long-term clinical success.

Figure 13a–d shows the dynamic reaction forces across different pore sizes of biomimetic Voronoi-latticed trabecular bone (VTB10, VTB15, VTB20, and VTB25), providing significant insight into the biomechanical performance of dental implant assemblies. The consistent lack of response forces for up to 0.23 s, followed by a rise in alignment with dynamic loading amplitudes, demonstrates the implants’ mechanical flexibility to masticatory stresses. Specifically, the research shows that VTB20 outperforms the other models due to its lower axial and buccolingual reaction forces and optimum mesiodistal response. This research implies that a pore size of 2.0 mm promotes a compromise between structural support and flexibility, allowing the implant to efficiently distribute stresses while maintaining its biomechanical integrity. The distinct mechanical responses observed at the 0.5 s mark across all models highlight the importance of pore size in tailoring the biomechanical properties of dental implants to mimic natural bone behavior, thereby improving the overall effectiveness and longevity of dental implant systems.

This work advances the biomechanical knowledge of bone–implant interactions by using advanced finite element analysis (FEA) that extends beyond typical unrealistic models. Our technique provides a more realistic representation of bone behavior in physiological settings by replicating the nonlinear and anisotropic features of biological tissues, as well as including dynamic loading conditions that replicate real-world pressures. This technique not only eliminates past shortcomings, but it also gives more in-depth insights into the performance and stability of biomimetic Voronoi lattice implants, making a substantial contribution to biomechanical engineering.

In summary, our results show that larger hole sizes result in a less dense, more open scaffold structure, boosting the surface complexity and perhaps improving osteointegration and mechanical bonding with bone tissue. An increased pore size increases the scaffold’s structural mimicry of the natural bone porosity, which has a major impact on the stress distribution and mechanical endurance of dental implants under dynamic conditions. A 2 mm pore size emerges as the best dimension, achieving a balance in stress distribution while also strengthening the structural integrity of both dental implants and retention screws. This adaptation emphasizes the importance of pore size in optimizing the biomechanical responses of biomimetic trabecular bone and dental implant assemblies to masticatory stresses, hence improving performance and durability.

## 5. Conclusions

This work sought to understand the biomechanical behavior of biomimetic Voronoi-latticed trabecular bone structures. Using finite element analysis (FEA) to evaluate the effect of altering pore sizes on stresses and micromotion magnitudes. Hence, the following are significant inferences from the present study:

- A direct relationship was discovered between the pore size and micromotion magnitude. This highlights the impact of pore size on the biomechanical behavior of biomimetic Voronoi trabecular bone. This relationship has significance during dental implant designs that optimize biomechanical performance.
- The research shows that the biomimetic Voronoi-latticed trabecular bone used in FEA has properties that are similar to real human cancellous bone when subjected to the applied external dynamic mastication stresses.
- The use of anisotropic characteristics in these novel biomimetic trabecular bones, as well as the simulation of dynamic mastication stresses, greatly improved the FEA findings. This method gives a more precise description of biomechanical interactions in the oral environment, resulting in more reliable implant designs.
- The study found that a 2.0 mm (VTB20) pore size presents the optimal integration of mechanical rigidity and minimum micromotion, making it a better option to further enhance the implant lifespan and performance.
- Micromotion optimization considerably reduces the chances of implant failure, such as fractures in implant components, bone resorption, and corrosion in a biofluid environment.

The research gives significant insight into the biomechanical optimization of dental implant designs by studying biomimetic Voronoi-latticed trabecular bones. This study expands possibilities for the design of dental implants that enhance biomechanical compatibility and performance by using modern FEA methods and considering the anisotropic properties of bone and dynamic loading scenarios. These results demonstrate the significance of using biomimetic approaches for developing and evaluating the performance of dental implants, in addition to making advancements in the fields of biomechanical engineering in dentistry.

## Figures and Tables

**Table 1:** FE mesh statistics of different biomimetic trabecular bone and implant component models.

	Crown		Abutment		Screw		Implant		Cortical Bone		Trabecular Bone		Total	
Bone Models	No. Element	No. Node	No. Element	No. Node	No. Element	No. Node	No. Element	No. Node	No. Element	No. Node	No. Element	No. Node	No. Element	No. Node
VTB10	107,954	20,670	85,243	17,463	627,098	121,624	160,676	31,737	146,767	31,180	1,936,663	584,049	3,064,401	806,723
VTB15	107,954	20,670	85,243	17,463	627,098	121,624	160,676	31,737	146,767	31,180	1,187,637	363,867	2,315,375	586,541
VTB20	107,954	20,670	85,243	17,463	627,098	121,624	160,676	31,737	146,767	31,180	791,391	248,917	1,919,129	471,591
VTB25	107,954	20,670	85,243	17,463	627,098	121,624	160,676	31,737	146,767	31,180	591,166	186,214	1,718,904	408,888

**Table 2:** Physical properties of materials for the FEA [32,35,37,47].

Materials	Young’s Modulus E (MPa)		Poisson’s Ratio ν		Density (g/cm^3^)	Strength (MPa)
Cortical bone *	E_x_	12,600	ν_xy_	0.3	1.79	190
	E_y_	12,600	ν_yz_	0.253		
	E_z_	19,400	ν_xz_	0.253		
			ν_yx_	0.3		
			ν_zy_	0.39		
			ν_zx_	0.39		
Cancellous bone *	E_x_	1148	ν_xy_	0.055	0.45	10
	E_y_	210	ν_yz_	0.01		
	E_z_	1148	ν_xz_	0.322		
			ν_yx_	0.01		
			ν_zy_	0.055		
			ν_zx_	0.322		
Gold abutment *	136,000		0.37		17.5	765
Porcelain *	68,900		0.28		2.44	145
Titanium grade 4 *	110,000		0.34		4.5	550

* The vectors of x, y and z are mean the infero-superior (Axial), mesiodistal, and buccolingual direction, respectively. And Implant & screw = Titanium grade 4, Abutment = Gold, Crown = Porcelain.

**Table 3:** Effects of pore size variation on structural parameters of biomimetic Voronoi lattice trabecular bone.

Symbolic Name	Pore Size (mm)	Beam Thickness (mm)	Randomization Seed	Average Beam Length (mm)	Lattice Beam Account	Lattice Node Count	Relative Density (%)	Surface-Area-to-Volume Ratio (%)
VTB10	1.0	0.4	250	0.4614	25,809	15,186	22.21	10.49
VTB15	1.5	0.4	250	0.5681	13,286	8182	15.19	11.58
VTB20	2.0	0.4	250	0.7532	5438	3535	8.84	12.21
VTB25	2.5	0.4	250	0.9203	3024	2058	6.27	12.4

## Data Availability

The original contributions presented in the study are included in the article, further inquiries can be directed to the corresponding author.
